# Supplementary material for: A Zika virus vaccine expressing premembrane-envelope-NS1 polyprotein
Source: Nat Commun. 2018 Aug 3;9:3067. doi: 10.1038/s41467-018-05276-4 (PMC6076265; doi:10.1038/s41467-018-05276-4)
Supplement: Supplementary file 1 — Supplementary Information [file 41467_2018_5276_MOESM1_ESM.pdf]

Supplementary information for

A novel Zika virus vaccine expressing pre-membrane, envelope, and NS1 proteins

* Corresponding author: li.926@osu.edu

Li et al

Supplementary Figure 1.

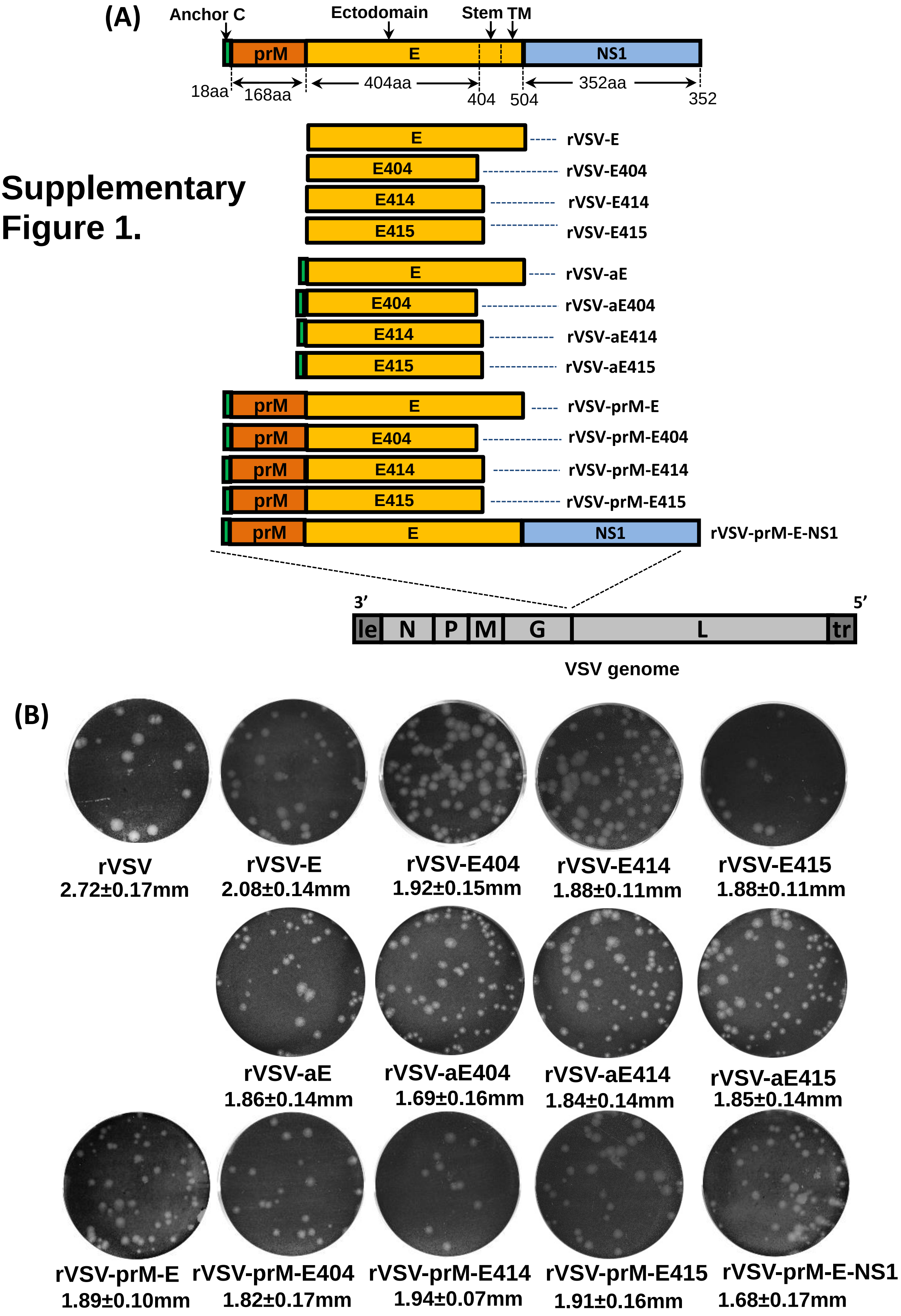

Supplementary Figure 1. Recovery of rVSV expressing ZIKV antigens. (A) Strategy to construct recombinant VSV expressing ZIKV antigens. **Top panel**: Schematic diagram of a polyprotein composed of anchor C (19 amino acids), pre-membrane (prM, 168 amino acids), envelope (E, 504 amino acids), and nonstructural protein 1 (NS1, 352 amino acids) was shown. **Middle panel**: The full-length ZIKV E, E truncations (E404, E414, and E415) lacking the stem and transmembrane (TM) domain, anchor C-E, anchor-E truncations (E404, E414, and E415), anchor C-prM-E, anchor C-prM-E truncations (prM-E404, prM-E414, and prM-E415), and anchor C-prM-E-NS1 genes were amplified from an infectious cDNA clone of ZIKV Cambodian strain by PCR, digested by XhoI and SmaI, and inserted into the same sites at the gene junction between G and L in the VSV genome. **Bottom panel**: The organization of nonsegmented negative-sense VSV genome is shown. Le, VSV leader sequence; N, nucleocapsid gene; P, phosphoprotein gene; M, matrix protein gene; G, glycoprotein gene; L, large polymerase gene; Tr, VSV trailer sequence. **(B) The plaque morphology of recombinant VSVs expressing ZIKV antigens**. Recombinant VSVs were recovered from an infectious cDNA clone. Plaque morphology of each recombinant virus was shown. All plaques were developed after 24 h of incubation. The diameter of a total of 10 plaques was measured for each recombinant virus. Data are expressed as mean ± standard deviation.

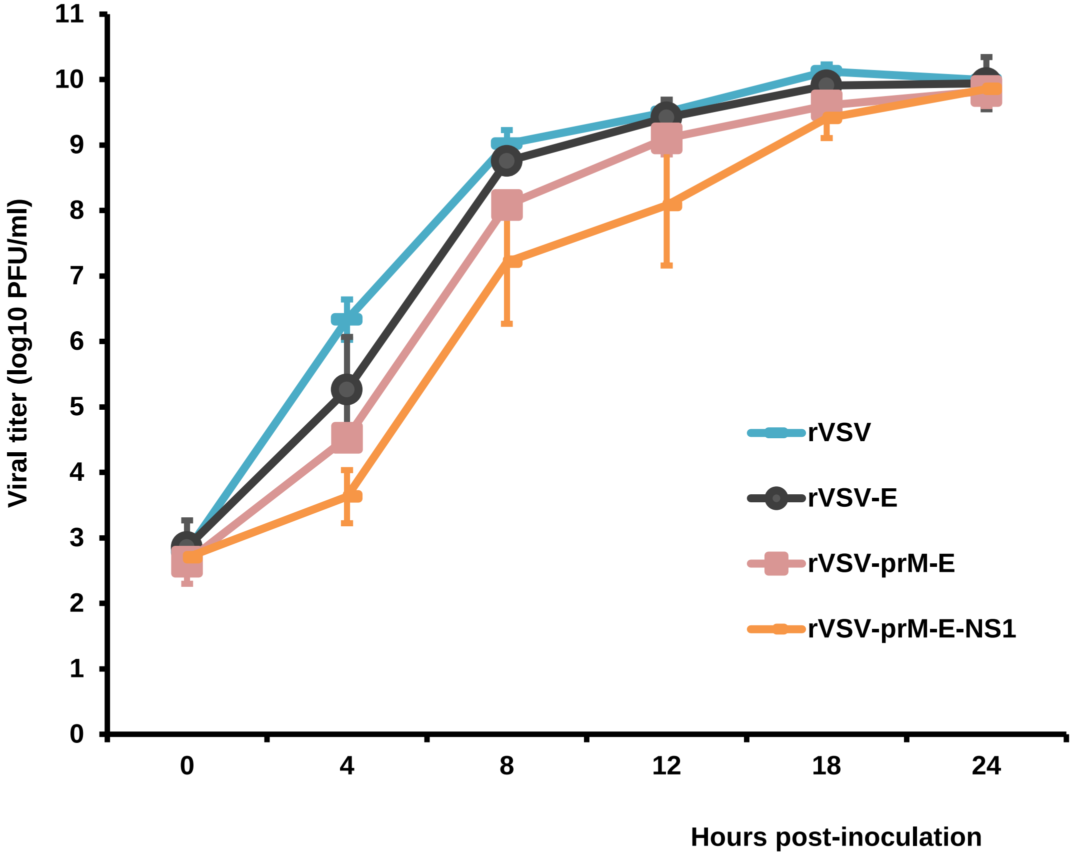

Supplementary Figure 2. Single-step growth curve of rVSV expressing ZIKV antigens. Confluent BSRT7 cells were infected with individual viruses at an MOI of 3.0. After 1 h of incubation, the inoculum was removed, the cells were washed with DMEM, and fresh medium (containing 2% fetal bovine serum) was added, followed by incubation at 37°C. Samples of supernatant were harvested at the indicated intervals over a 24-h time period, and the viral titer was determined by plaque assay. Data are GMT of three independent experiments ± standard deviation. The *P* values of rVSV-prM-E-NS1 compared to other recombinant viruses at 4, 8, 12, 18, and 24 h are 0.0098, 0.0045, 0.0025, 0.02, and 0.694, respectively, demonstrating that rVSV-prM-E-NS1 had a significant delay compared to other viruses.

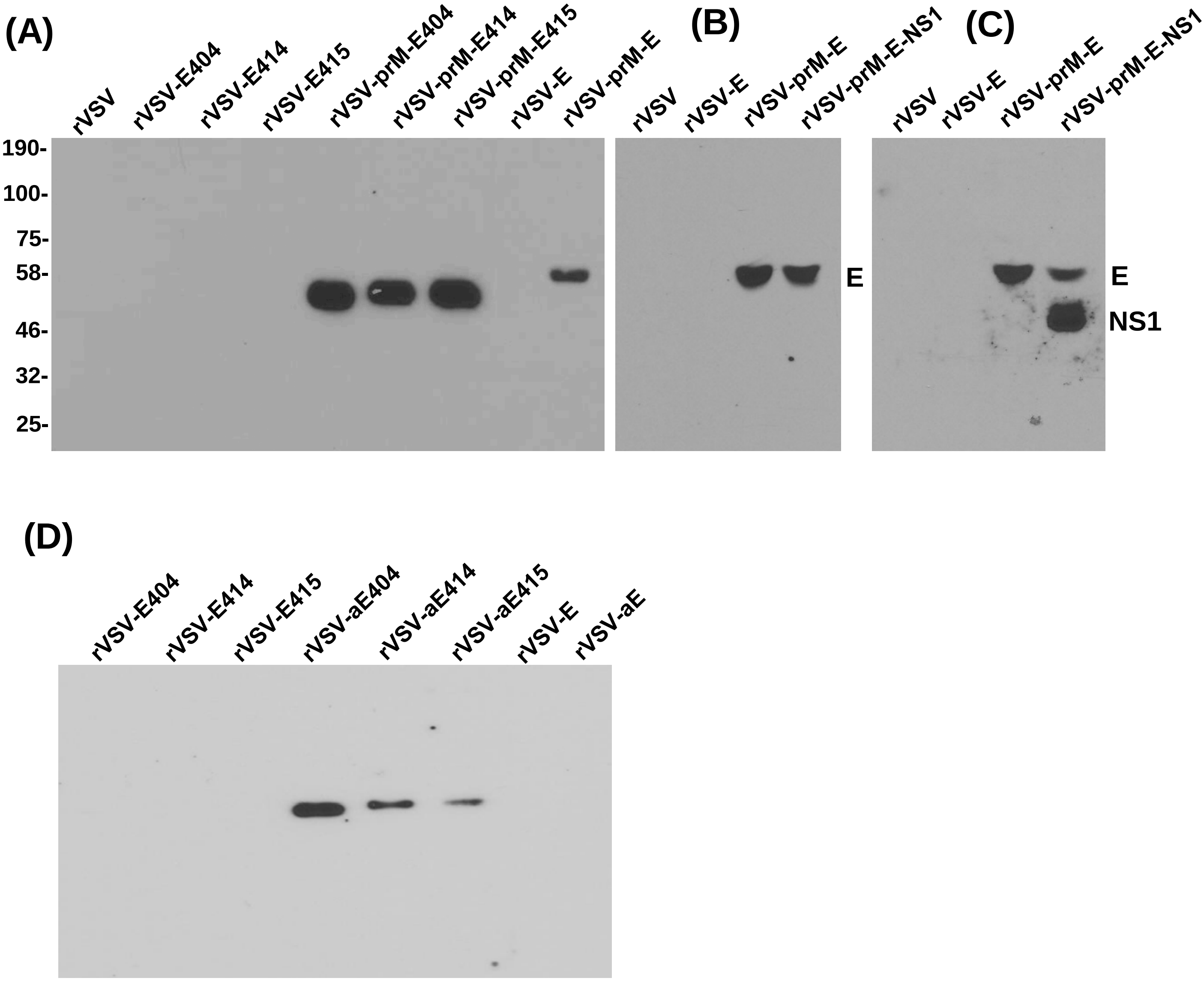

Supplementary Figure 3. Detection of ZIKV E and NS1 proteins in cell culture supernatants. BSRT7 cells were infected with each recombinant virus expressing ZIKV antigen at an MOI of 3.0. At 24 h post-inoculation, cell culture supernatants were harvested, and 10 µl of supernatants were analyzed by Western blot using E specific antibody (**A, B, and D**) or ZIKV serum antibody (**C**).

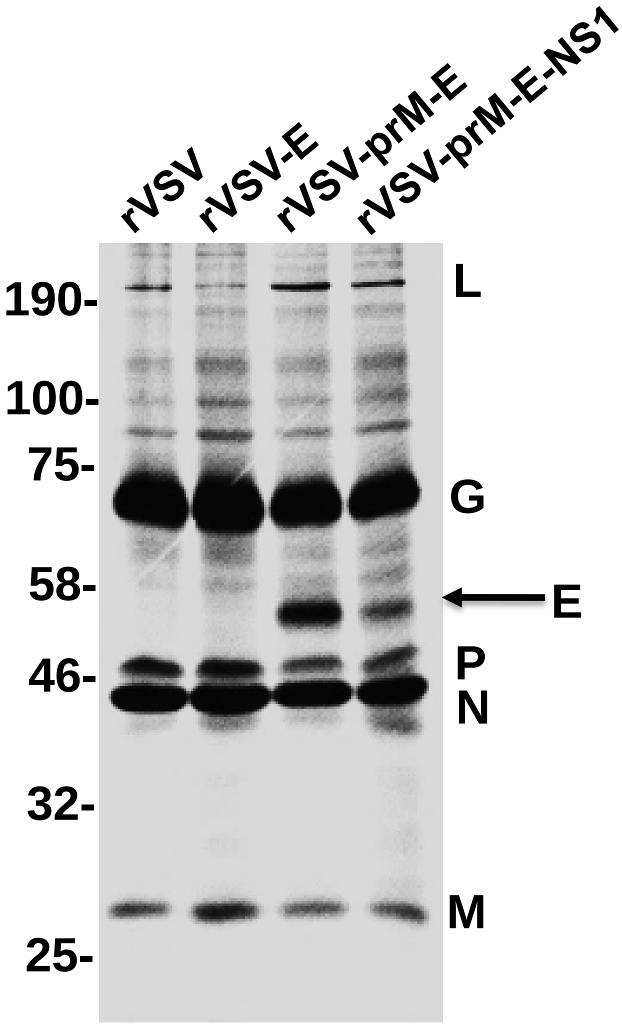

Supplementary Figure 4. Detection of ZIKV E protein expression using [^{35}S]methionine labelling. BSRT7 cells were infected with each recombinant virus at an MOI of 20. After 3 h post-infection, proteins were metabolically labeled by incorporation of [^{35}S]methionine-cysteine in the presence of actinomycin D. After 4 h of incubation, cytoplasmic extracts were harvested, and proteins were analyzed by SDS-PAGE and detected by using a phosphorimager. The identities of VSV proteins are shown on the right.

Supplementary Figure 5

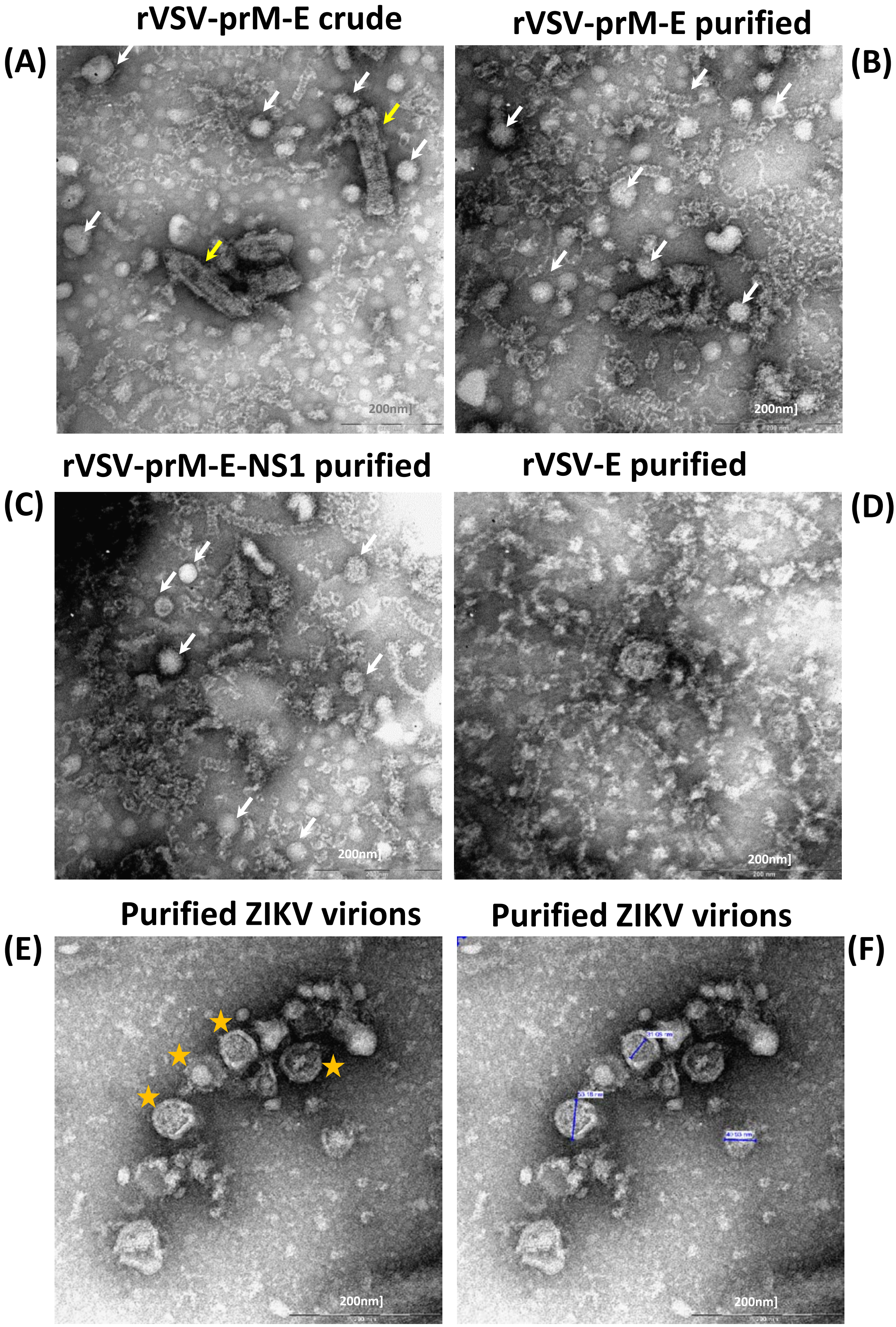

Supplementary Figure 5. Electron microscopy analysis of ZIKV virus-like particles (VLPs). ZIKV VLPs were negatively stained with 1% ammonium molybdate and visualized by a transmission electron microscope. rVSV-prM-E crude indicates a mixture of ZIKV VLPs and VSV virions from supernatant harvested from BSRT7 cells infected by rVSV-prM-E. ZIKV VLPs were further purified from rVSV-prM-E or rVSV-prM-E-NS1-infected cells. No VLPs were found in rVSV-E-infected cells.

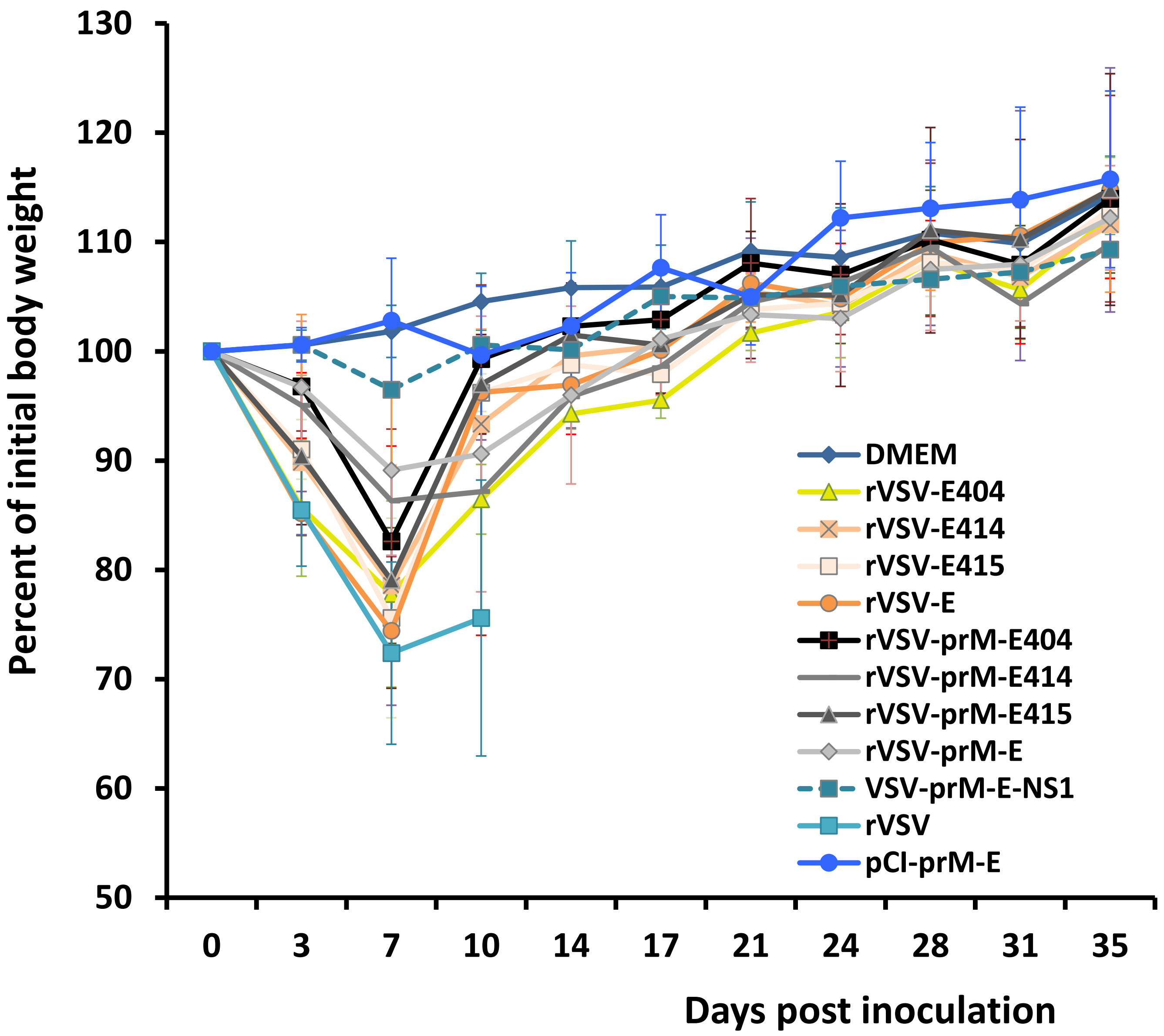

Supplementary Figure 6. Dynamics of mouse body weight after inoculation with rVSV expressing ZIKV antigen. Five female BALB/c mice in each group were intranasally inoculated with 10^6 PFU of rVSV or rVSV expressing ZIKV antigens. For DNA vaccine, mice were vaccinated intramuscularly with 50 µg of pCI-prM-E, and were boosted with the same dose two weeks later. The body weight for each mouse was evaluated at indicated time points. The average body weights of five mice ± standard deviation were shown. At day 7, two out of five mice were dead in the rVSV group. The remaining three mice were dead at day 10.

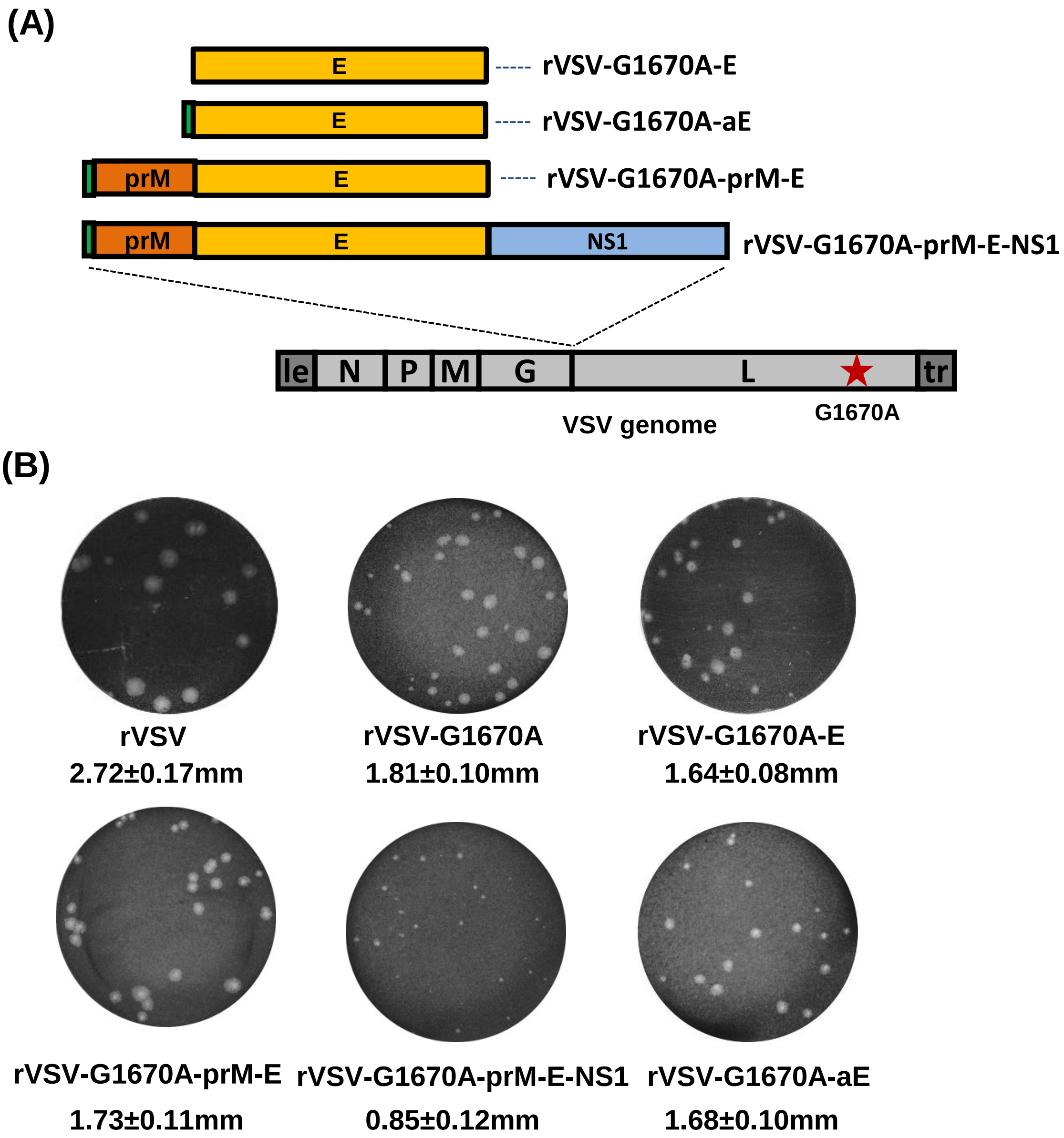

Supplementary Figure 7. Recovery of MTase-defective rVSV (mtd-rVSV) expressing ZIKV antigens. (A) Strategy to construct mtd-rVSVs. A single point mutation (G1670A) in the SAM binding site in the L protein was introduced into the VSV backbone. The ZIKV E, aE, prM-E, or prM-E-NS1 was inserted at the gene junction between G and L genes. (B) Plaque morphology of each recombinant virus is shown. All plaques were developed after 24 h of incubation. The diameter of a total of 10 plaques from three independent plaque assays was measured for each recombinant virus. Data are expressed as mean ± standard deviation.

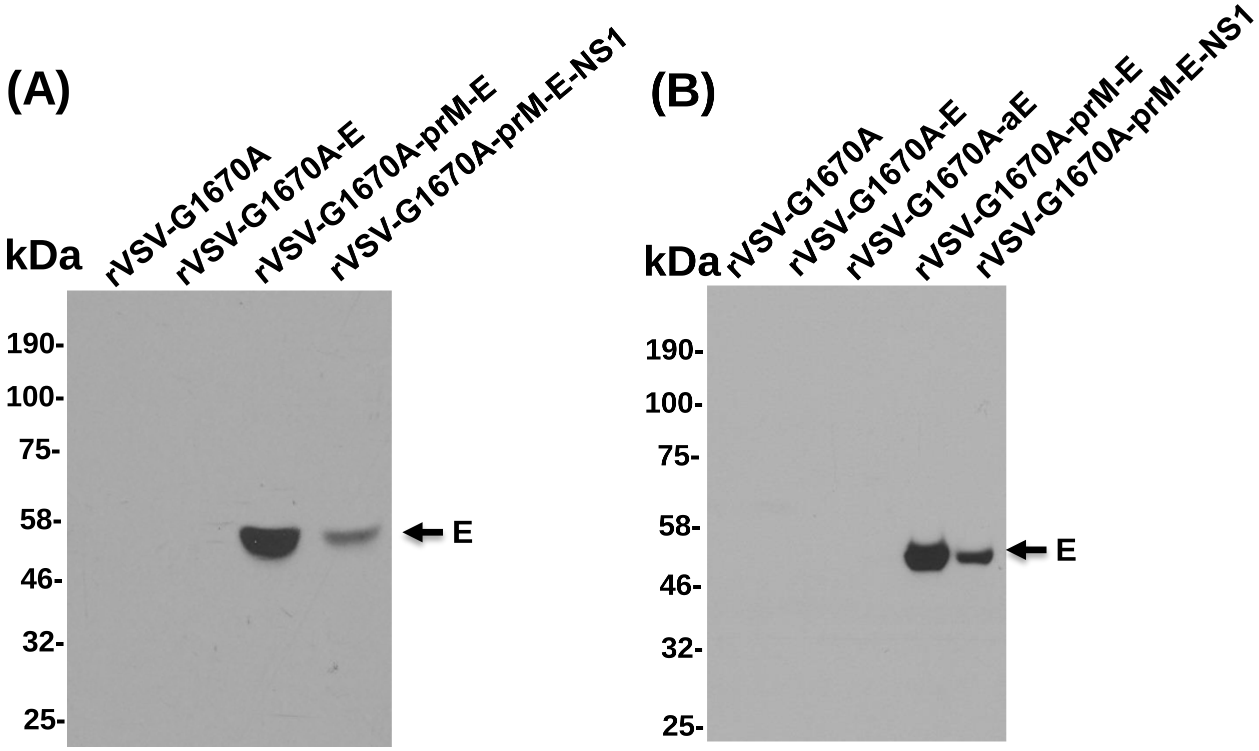

Supplementary Figure 8. Detection of ZIKV E protein in cell culture supernatants. BSRT7 cells were infected with each recombinant virus expressing ZIKV antigen at an MOI of 3.0. At 36 h post-inoculation, cell culture supernatants were harvested, and 10 µl of supernatants were analyzed by Western blot using E specific antibody.

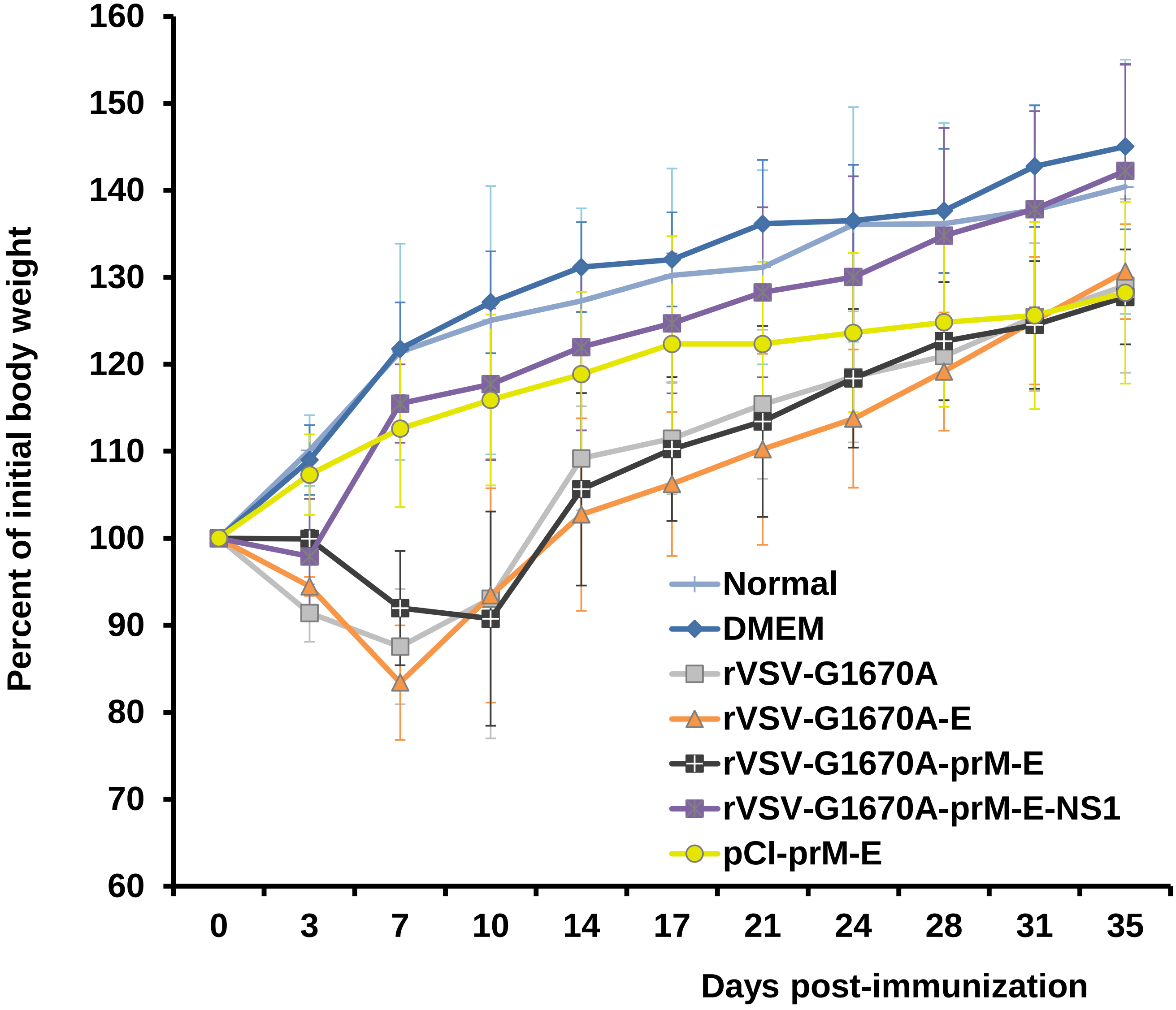

Supplementary Figure 9. Dynamics of body weight change of BALB/c mice after immunization with mtdVSV-based vaccine candidates. Ten 4-week-old BALB/c mice (5 female and 5 male) in each group were intranasally inoculated with 10^6 PFU of each mtdVSV expressing ZIKV antigens. For DNA vaccine, mice were vaccinated intramuscularly with 50 µg of pCI-prM-E and were boosted with same dose two weeks later. Mouse body weight was monitored every 3-4 days until day 35 post-immunization. The average body weights of ten mice ± standard deviation in each group are shown.

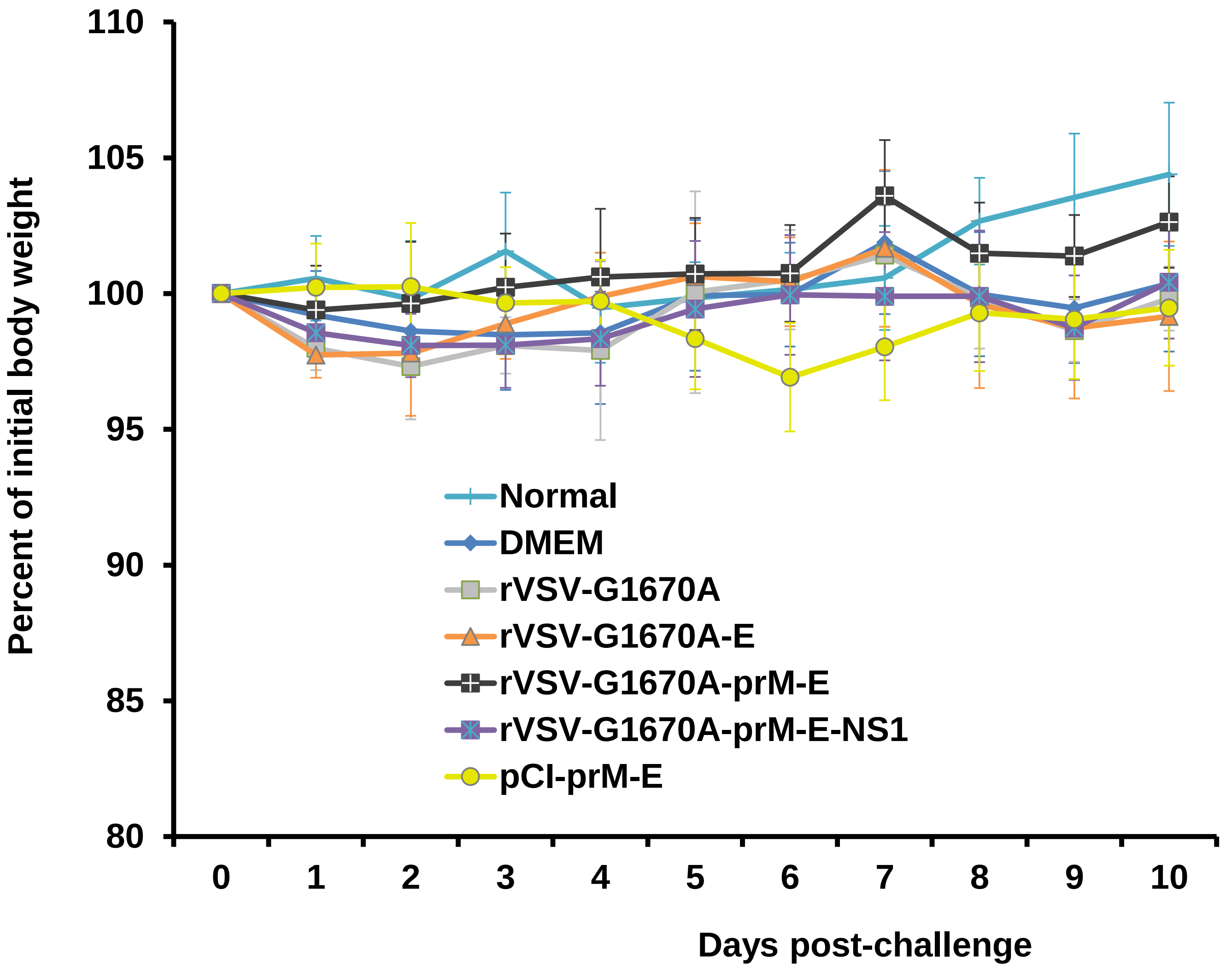

Supplementary Figure 10. Dynamics of body weight change of BALB/c mice after challenge with ZIKV. Ten four-week BALB/c mice (5 female and 5 male) in each group were intranasally inoculated with 10^6 PFU of each mtdVSV expressing ZIKV antigens. For DNA vaccine, mice were vaccinated intramuscularly with 50 µg of pCI-prM-E and were boosted with same dose two weeks later. At week 5, mice were administered 1.8 mg of anti-ifnar1 blocking antibody and 24 h later challenged with 10^5 PFU of ZIKV Cambodian strain via the I.V. route. After challenge, the body weight for each mouse was evaluated at indicated time points. The average body weights of ten mice ± standard deviation were shown. No significant difference in body weight was observed among groups (*P*>0.05, t-test).

Supplementary Figure 11.

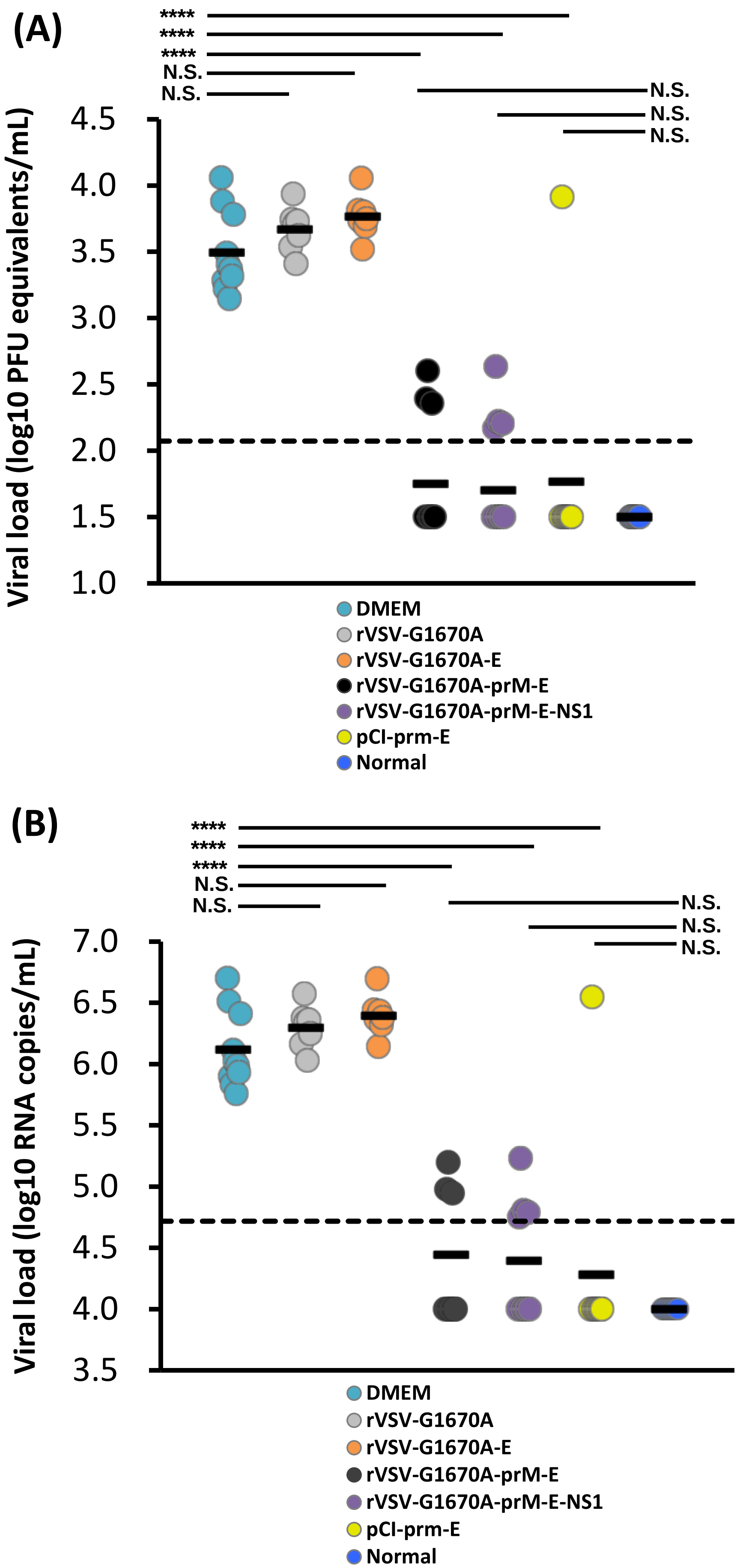

Supplementary Figure 11. mtdVSV-based vaccine protects BALB/c mice from viremia at days 3 post-challenge. The viremia data at days 3 from Fig.4A were expressed as a different format, showing the numbers of mice in each group were above the detection limit. The level of viremia was measured by real-time RT-PCR and expressed as PFU equivalent RNA/ml **(A)** or exact RNA copies/ml **(B)**. Data were GMT of 10 mice (black bar). Exact *P* value (t-test) from top to bottom in each panel: (A) **** $P = 2.81 \times 10^{-5}$, **** $P = 1.30 \times 10^{-7}$, **** $P = 2.63 \times 10^{-7}$; (B) **** $P = 2.81 \times 10^{-5}$, **** $P = 1.30 \times 10^{-7}$, **** $P = 2.63 \times 10^{-7}$. N.S. indicates not significant.

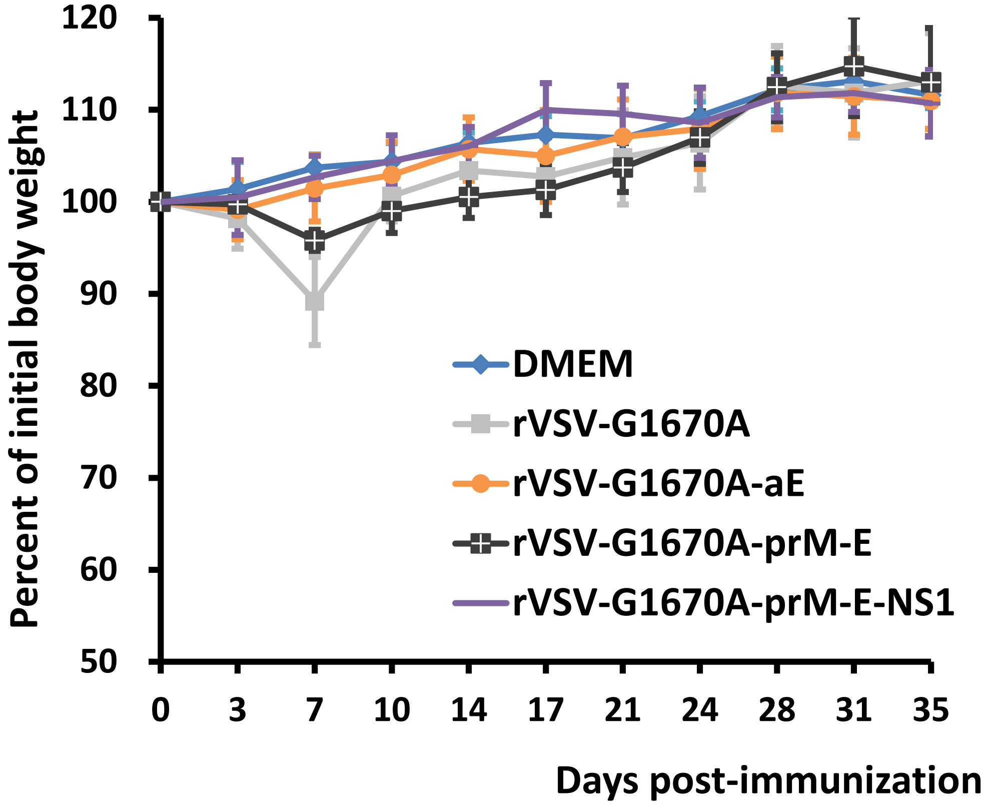

Supplementary Figure 12. Dynamics of body weight change of BALB/c mice after immunization with mtdVSV-based vaccine candidates. Five six-week-old female BALB/c mice in each group were intranasally inoculated with 10^6 PFU of each mtdVSV expressing ZIKV antigens. Mouse body weight was monitored until day 35 post-immunization. Data shown are mean of 5 mice ± standard deviation.

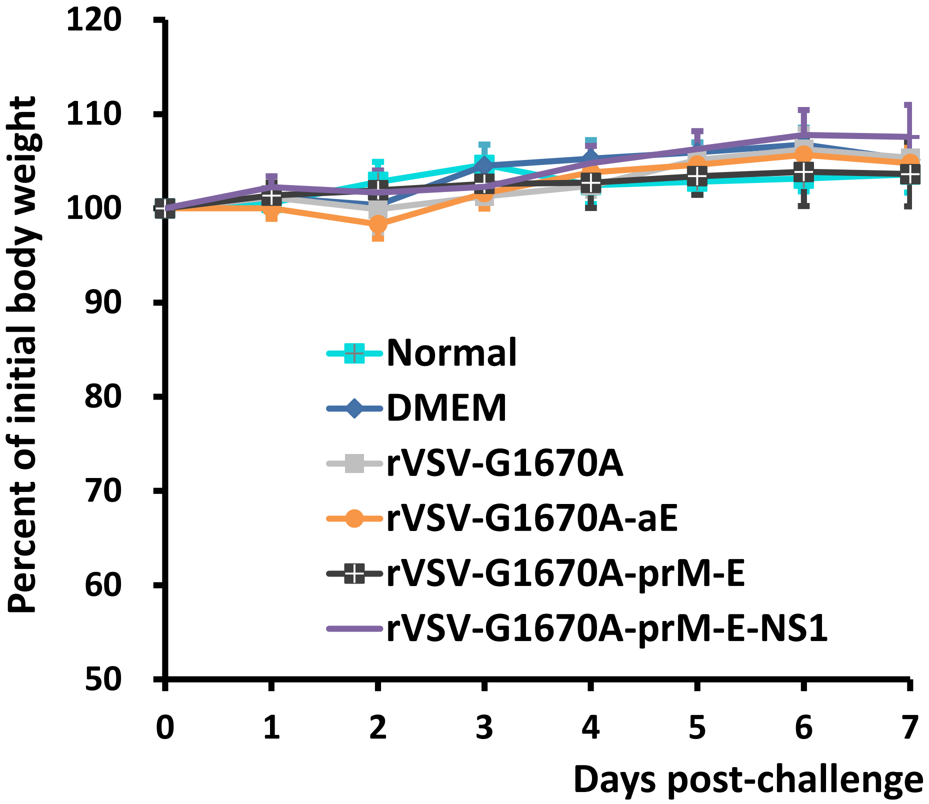

Supplementary Figure 13. Dynamics of body weight change of BALB/c mice after challenge with ZIKV. Mice from Fig.S11 were challenged with 10^5 PFU of ZIKV Cambodian strain. After challenge, the body weight for each mouse was evaluated until day 7 post-challenge. Data shown are mean of 5 mice ± standard deviation.

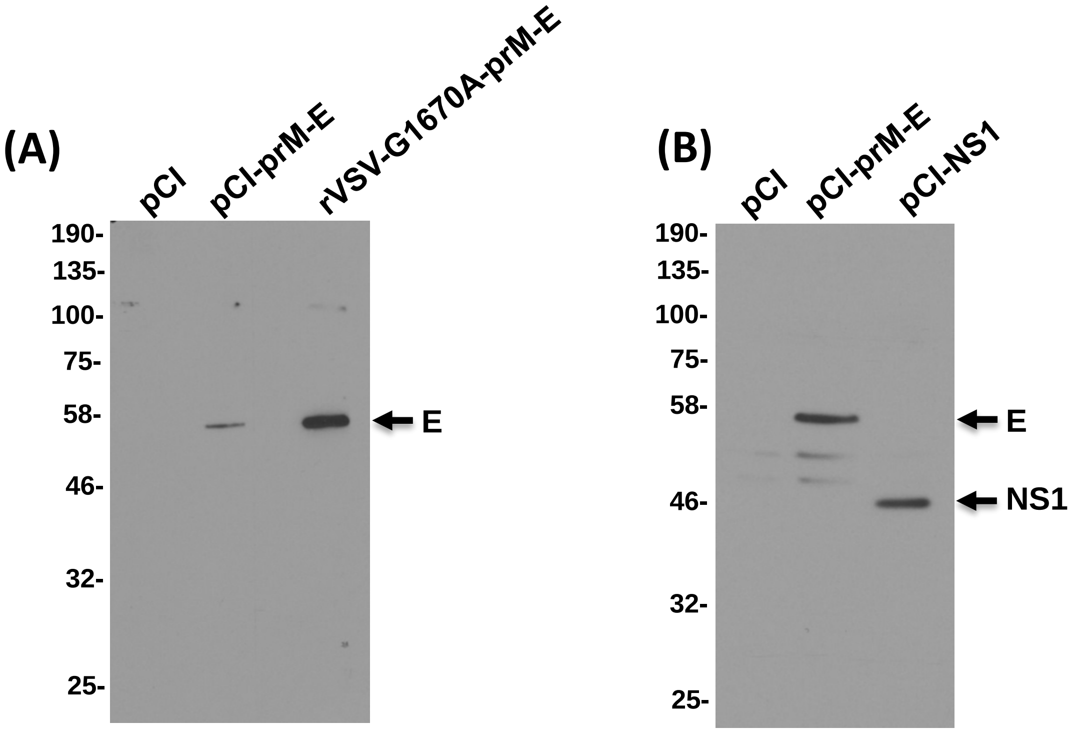

Supplementary Figure 14. Expression of ZIKV E and NS1 proteins by DNA vaccine constructs. **(A) Comparison of E protein expression by pCI-prM-E and rVSV-G1670A-prM-E.** BSRT7 cells were transfected with 4 µg of pCI-prM-E or infected with rVSV-G1670A-prM-E at an MOI of 1.0. Cell lysates were harvested at 24 h post-transfection or post-infection, and analyzed by Western blot using anti-ZIKV E monoclonal antibody. **(B) Expression of NS1 protein by pCI-NS1.** HEK293T cells were transfected with 4 µg of pCI-NS1 or pCI-prM-E. Cell lysates were harvested at 48 h post-transfection, and analyzed by Western blot using antibody against ZIKV E or NS1 protein.

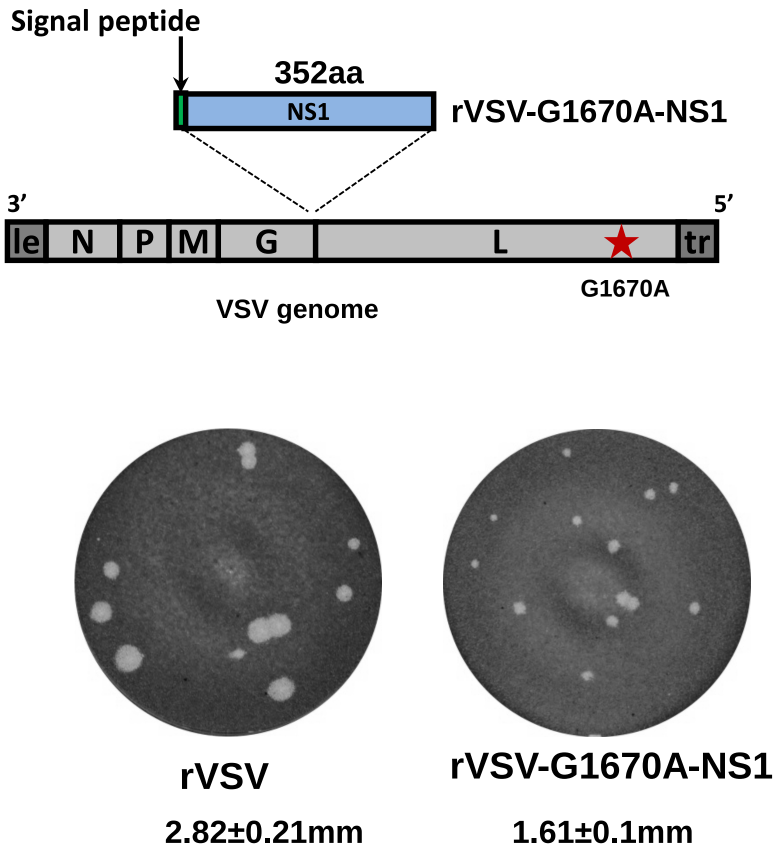

Supplementary Figure 15. Recovery of MTase-defective rVSV (mtd-rVSV) expressing ZIKV NS1 alone. (A) Strategy to construct mtd-rVSVs. A single point mutation (G1670A) in the SAM binding site in the L protein was introduced into the VSV backbone. The ZIKV anchor C-NS1 gene was inserted at the gene junction between the G and L genes. **(B) Plaque morphology of each recombinant virus.** All plaques were developed after 24 h of incubation. The diameter of a total of 10 plaques was measured for each recombinant virus. Data are expressed as mean ± standard deviation.

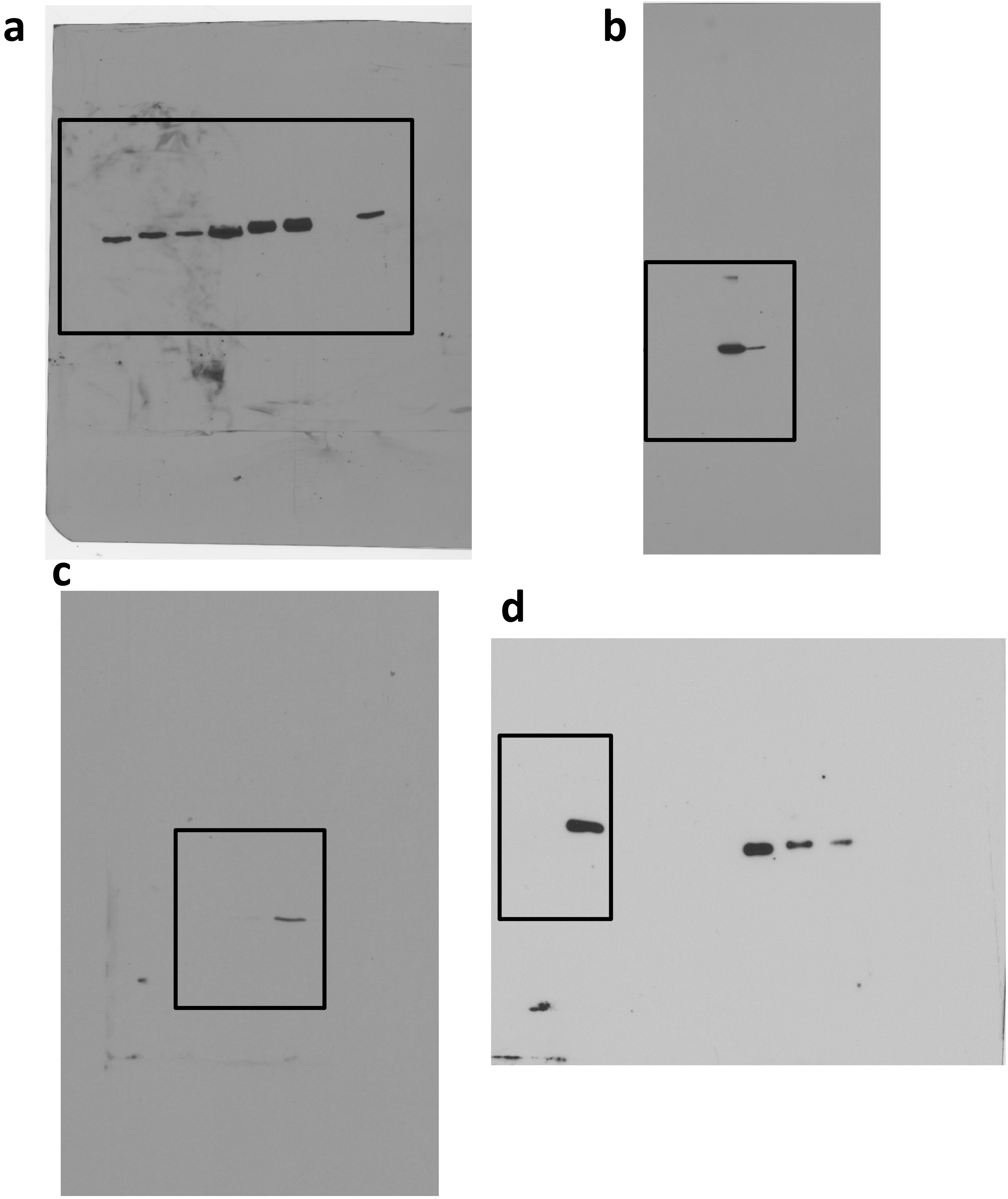

Supplementary Figure 16. Uncropped images of Western blots. (a) For Fig. 1A. (b) For Fig. 1B. (c) For Fig. 1C. (d) For Fig. 1d left.

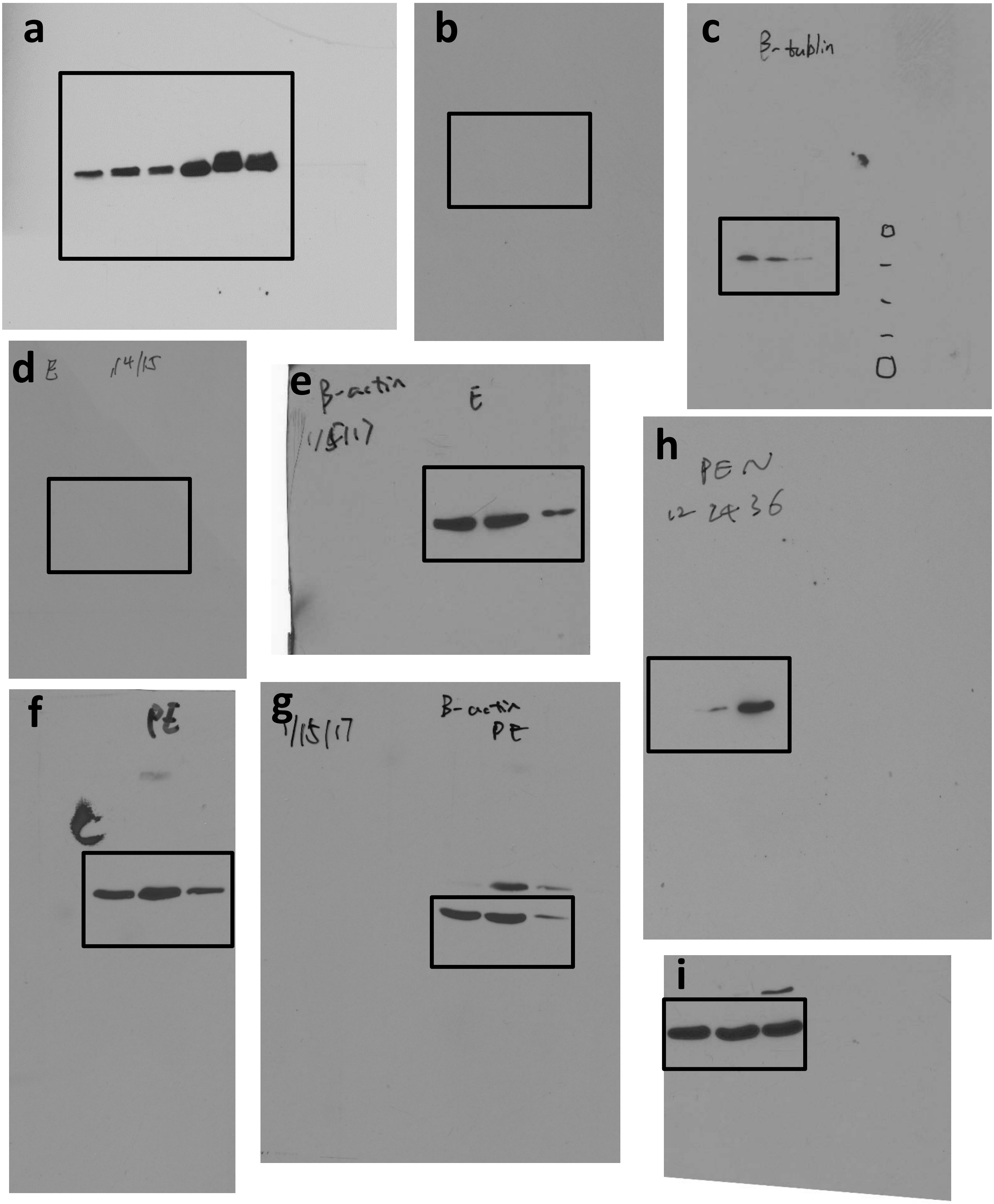

Supplementary Figure 17. Uncropped images of Western blots. (a) For Fig. 1D right. (b) For Fig. 1E left-up, E. (c) For Fig. 1E-left-up, actin. (d) For Fig. 1E-left-down, E. (e) For Fig. 1E-left-down, actin. (f) For Fig. 1E right-up, E. (g) For Fig. 1E-right-up, actin. (h) For Fig. 1E-right-down, E. (i) For Fig. 1E-right-down, actin.

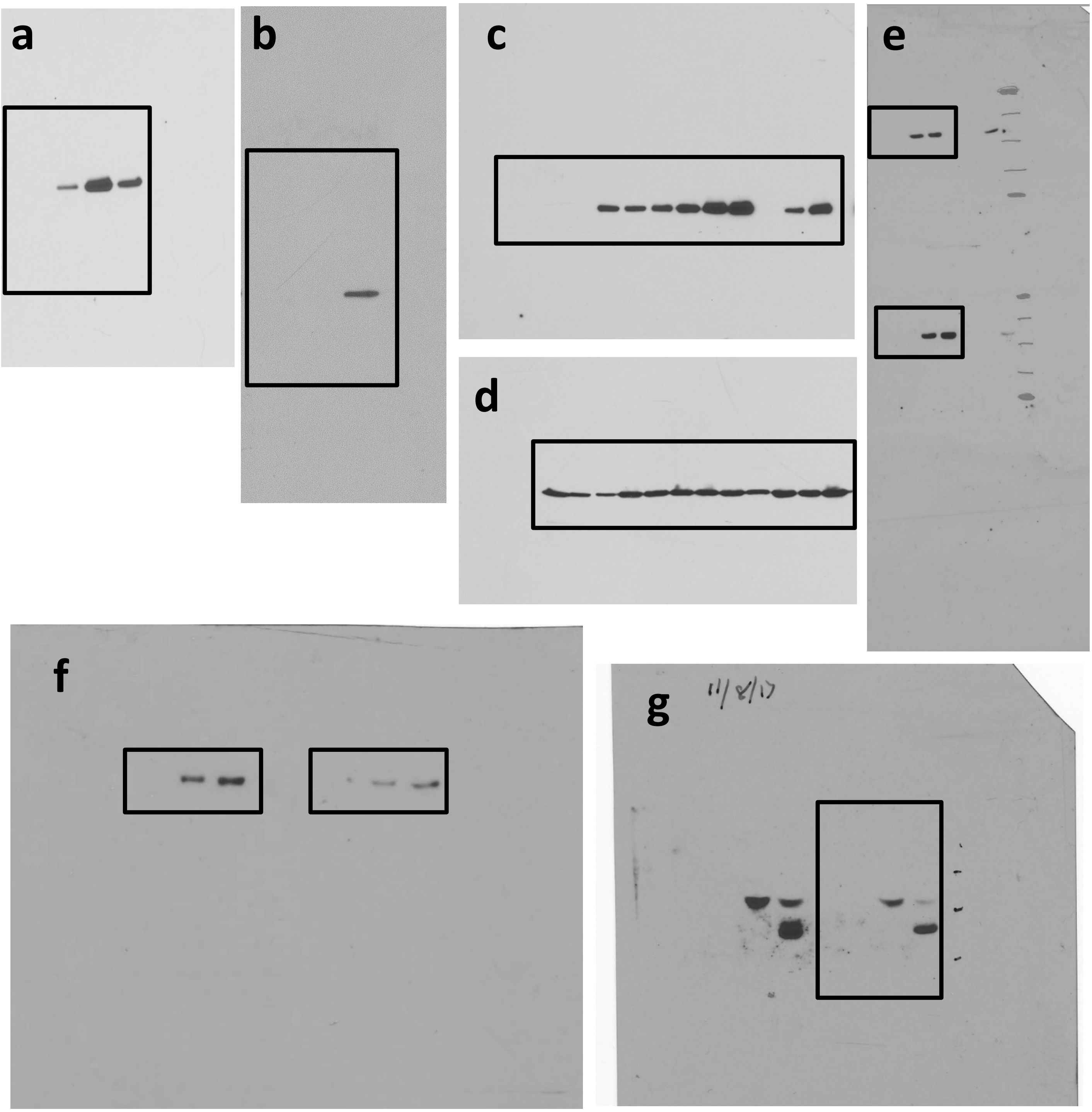

Supplementary Figure 18. Uncropped images of Western blots. (a) For Fig. 2B left. (b) For Fig. 2B right. (c) For Fig. 2C up. (d) For Fig. 2C down. (e) For Fig. 2D left. (f) For Fig. 2D right. (g) For Fig.2E.

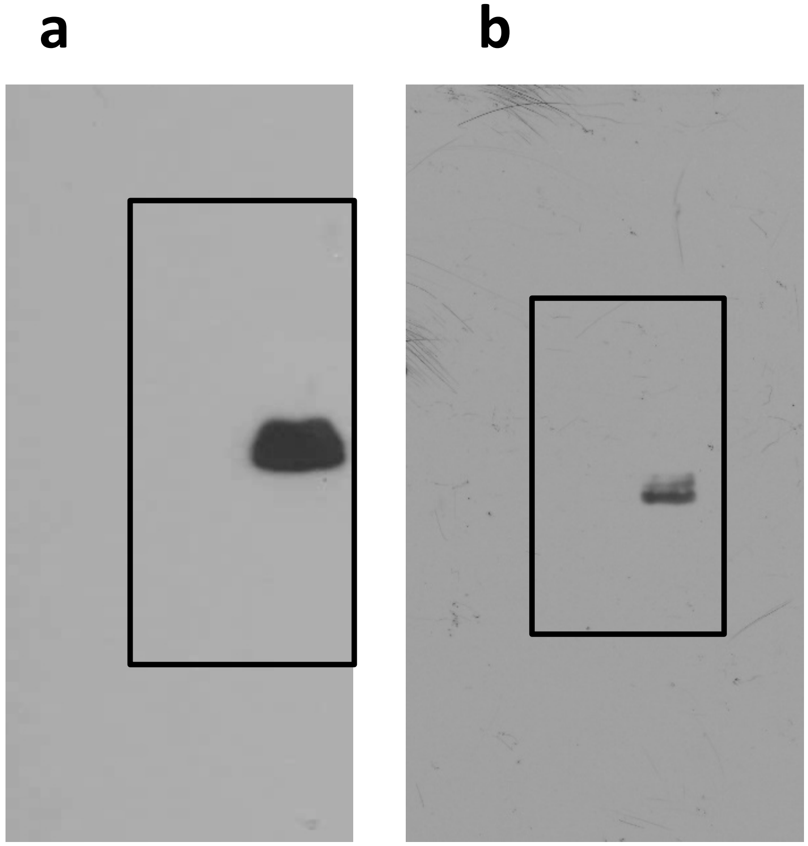

Supplementary Figure 19. Uncropped images of Western blots. (a) For Fig. 6D left. (b) For Fig. 6D right.

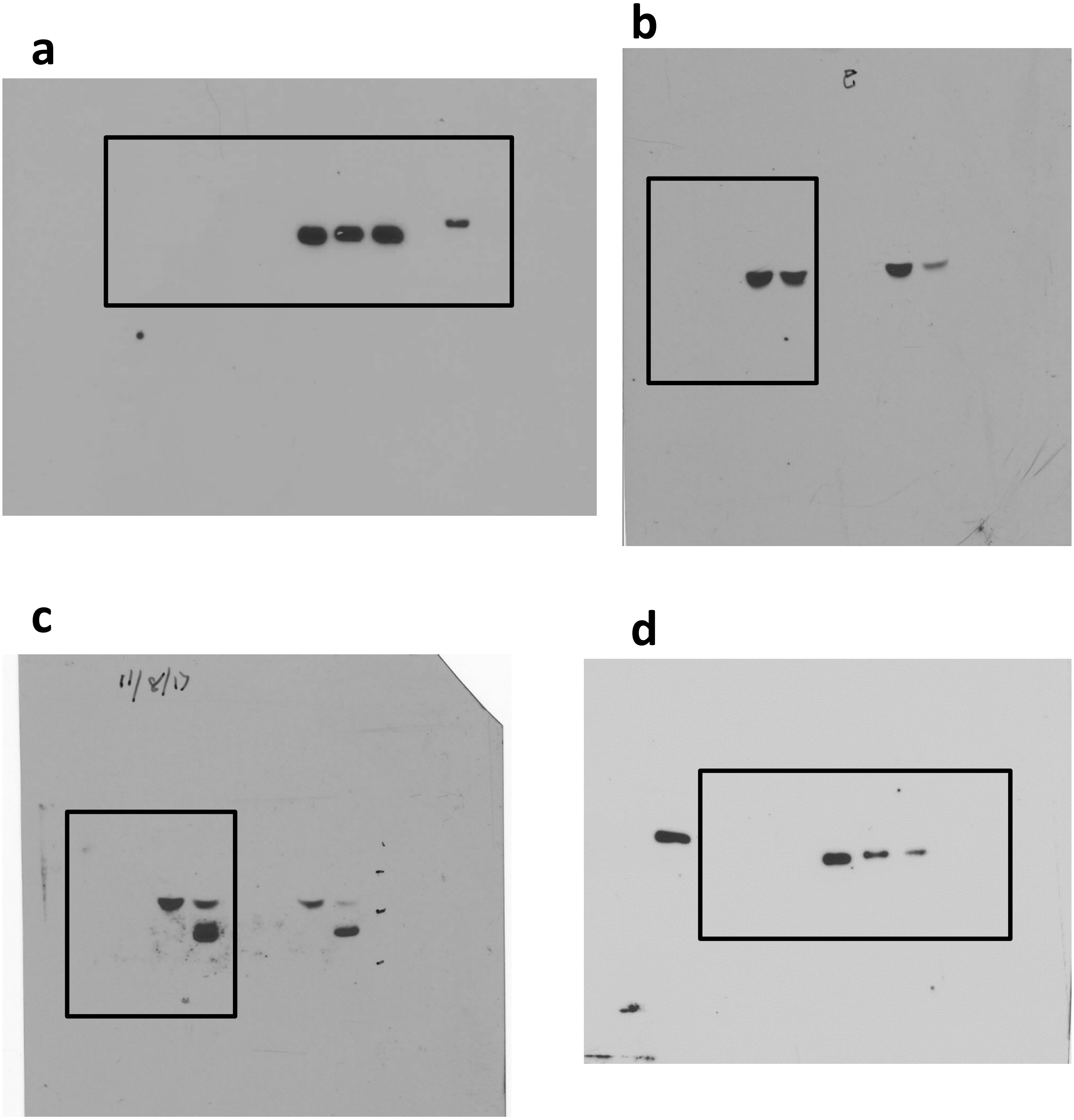

Supplementary Figure 20. Uncropped images of Western blots. (a) For supplementary Figure 3A. (b) For supplementary Figure 3B. (c) For supplementary Figure 3C. (d) For supplementary Figure 3D.

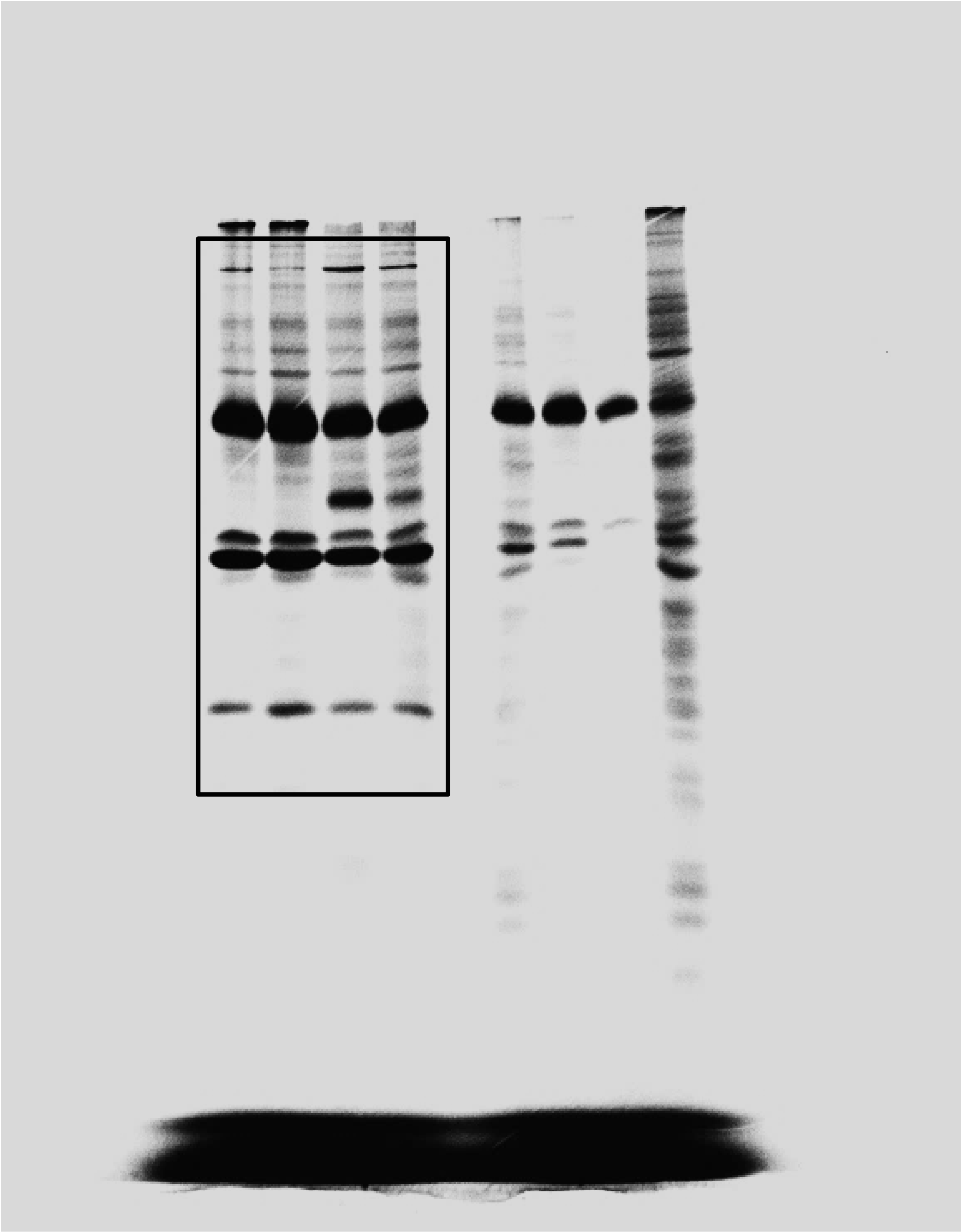

Supplementary Figure 21. Uncropped images of SDS-PAGE gel. For supplementary Figure 4

a **b**

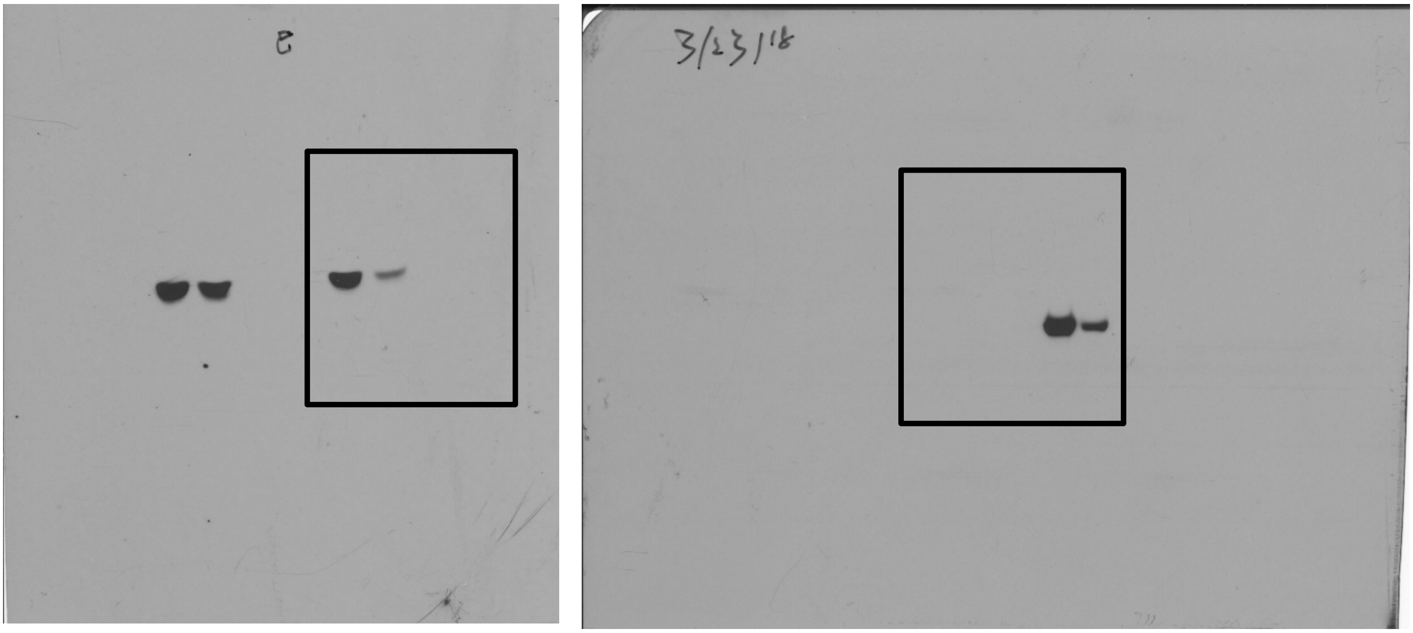

Supplementary Figure 22. Uncropped images of Western blots. (a) For supplementary Figure 8A. (b) For supplementary Figure 8B.

a b

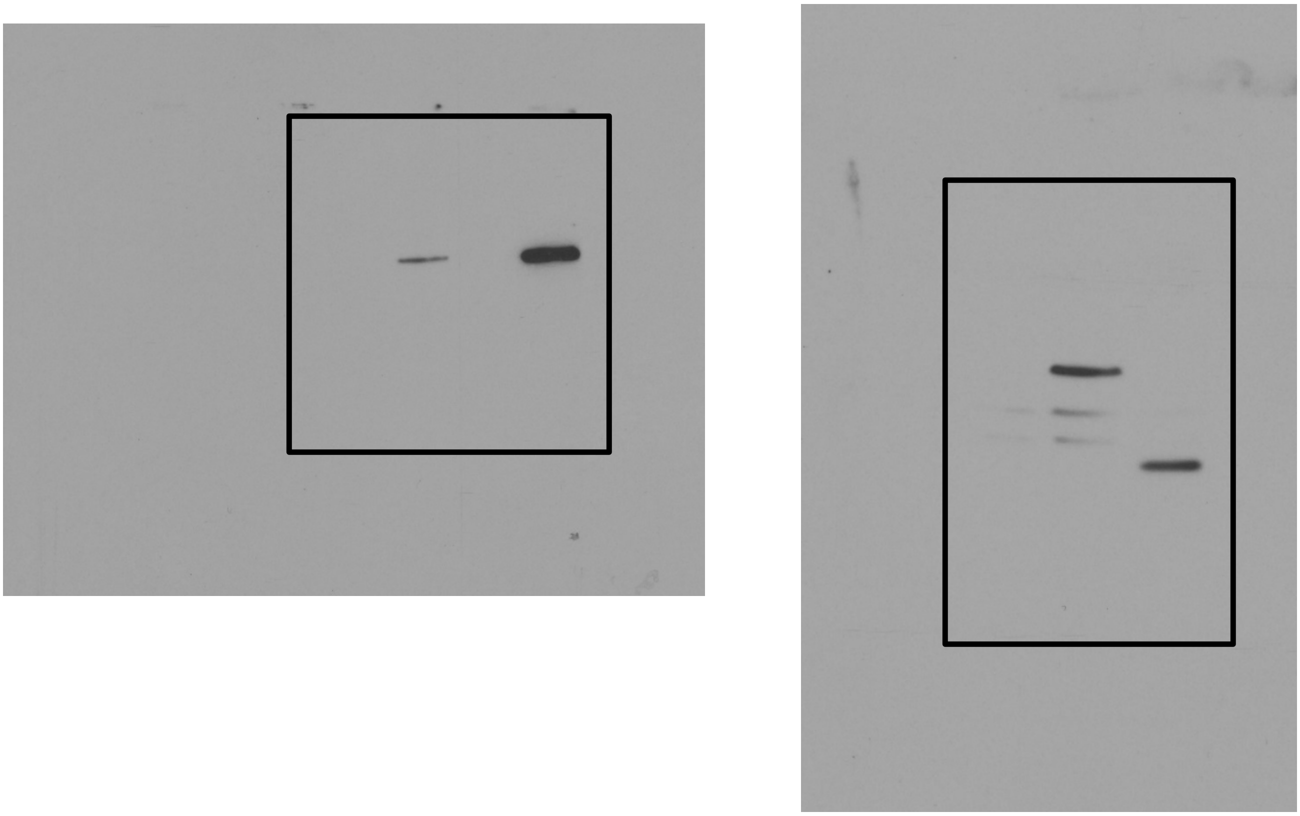

Supplementary Figure 23. Uncropped images of Western blots. (a) For supplementary Figure 14A. (b) For supplementary Figure 14B.

Supplementary Table 1

Summary of attenuation, antibody response, and protection efficacy of VSV constructs

Constructs	Attenuation [A]		E-specific antibody responses [B]		NS1-specific antibody responses [C]		Protection [D]	
	BALB/c	A129	BALB/c	A129	BALB/c	A129	BALB/c	A129
**rVSV**	Virulent	Virulent	0	ND	ND	ND	ND	ND
**rVSV-E**	Moderate	ND	2.00	ND	ND	ND	ND	ND
**rVSV-E404**	Moderate	ND	2.70	ND	ND	ND	ND	ND
**rVSV-E414**	Moderate	ND	2.80	ND	ND	ND	ND	ND
**rVSV-E415**	Moderate	ND	2.75	ND	ND	ND	ND	ND
**rVSV-prM-E404**	Moderate	ND	3.57	ND	ND	ND	ND	ND
**rVSV-prM-E414**	Moderate	ND	4.01	ND	ND	ND	ND	ND
**rVSV-prM-E415**	Moderate	ND	4.53	ND	ND	ND	ND	ND
**rVSV-prM-E**	Moderate	ND	3.81	ND	ND	ND	ND	ND
**rVSV-prM-E-NS1**	High	Virulent	2.66	ND	ND	ND	ND	ND
**pCI-prM-E**	NA	NA	3.32	3.14	ND	0	Complete	Complete
**rVSV-aE**	ND	ND	ND	ND	ND	ND	ND	ND
**rVSV-aE404**	ND	ND	ND	ND	ND	ND	ND	ND
**rVSV-aE414**	ND	ND	ND	ND	ND	ND	ND	ND
**rVSV-aE415**	ND	ND	ND	ND	ND	ND	ND	ND
**rVSV-G1670A**	Moderate	ND	0	ND	0	ND	No protection	ND
**rVSV-G1670A-E**	Moderate	ND	0	ND	0	ND	No protection	ND
**rVSV-G1670A-aE**	Moderate to high	ND	2.30	ND	0	ND	No protection	ND
**rVSV-G1670A-prM-E**	Moderate to high	Moderate	3.26	2.60	0	0	Complete	Complete
**rVSV-G1670A-prM-E-NS1**	High	High	2.66	3.51	3.75	4.35	Complete	Complete
**rVSV-G1670A-NS1**	Moderate to high	ND	0	ND	5.37	ND	Partial	ND
**pCI-NS1**	NA	NA	0	0	3.87	0.74	Partial	Partial
**pCI**	NA	NA	0	0	0	0	No protection	No protection

A: “Virulent” indicates that construct causes severe VSV clinical signs and mortality; “moderate” indicates that constructs cause mild illness, 10-20% weight loss, and recovered; “high” indicate that constructs cause no VSV clinical sign, no or less than 5% weight loss. NA = Not Applicable; ND = Not Done.

B: Log_{10} E-specific ELISA antibody at week 5 (BALB/c) or 3 (A129) post-vaccination was indicated in the column. DNA vaccine received booster vaccination whereas all VSV constructs were single dose vaccination.

C: Log_{10} NS1-specific ELISA antibody at week 5 post-vaccination was indicated in the column. DNA vaccine received booster vaccination whereas all VSV constructs were single dose vaccination.

D: “Complete” indicates that mice lack ZIKV clinical sign and viremia after ZIKV challenge. “Partial” indicates that some mice exhibit clinical sign or viremia after ZIKV challenge but lower than unvaccinated challenged control. “No protection” indicates that mice exhibit the same level of clinical sign or viremia as the unvaccinated challenged control.
